# A case of high-grade non-intestinal paranasal sinus adenocarcinoma primary in the maxillary sinus: targeted therapy after postoperative immunocombination with chemotherapy

**DOI:** 10.1007/s00432-024-05744-z

**Published:** 2024-08-03

**Authors:** Lu Yang, Lu Lu, Ji Ma, Zaihua Xu, Na Li

**Affiliations:** 1 Department of Oncology, Suining Central Hospital, Suining, China; 2 Department of Radiotherapy, Friendship Hospital of ILY Kazak Autonomous Prefecture, Xinjiang, China

**Keywords:** High-grade non-intestinal-type sinonasal adenocarcinoma, Immunotherapy, Targeted therapy

## Abstract

**Background:**

High-grade non-intestinal-type sinonasal adenocarcinoma (non-ITAC) is a rare and aggressive form of adenocarcinoma with poor prognosis. The current standard treatment approach involves surgery combined with radiation therapy. However, there is a need for exploring additional treatment modalities to improve patient outcomes.

**Case presentation:**

We present a case of a 65-year-old male patient who presented with pain in the right maxillary sinus and was diagnosed with high-grade non-ITAC following surgery. Postoperative pathology revealed tumor invasion into bone tissue and vascular invasion, necessitating further treatment. The patient underwent radiation therapy, followed by immunotherapy with carilizumab combined with chemotherapy. During the maintenance immunotherapy period, tumor progression was observed, and genetic testing identified EGFR and TP53 mutations. Consequently, the patient was treated with gefitinib, a targeted therapy drug. Notably, the patient’s lung metastases showed a gradual reduction in size, indicating a favorable treatment response. The patient is currently undergoing oral treatment with gefitinib.

**Conclusions:**

This case report highlights the potential benefit of combining immunotherapy and targeted therapy in the treatment of high-grade non-ITAC. Despite the rarity of this cancer type, this approach may offer an alternative treatment strategy for patients with this aggressive disease. We hope that this case can contribute to a deeper understanding of high-grade non-ITAC and promote the application of immunotherapy and targeted therapy in improving survival rates for patients with this condition.

## Introduction

In the 5th edition of the World Health Organization (WHO) classification, primary sinonasal adenocarcinoma is divided into two subtypes: intestinal-type sinonasal adenocarcinoma (ITAC) and non-intestinal-type sinonasal adenocarcinoma (non-ITAC) (Thompson et al. 2022).Non-ITAC neither morphologically exhibits intestinal features nor salivary features, and is rarer than ITAC (Purgina et al. 2015a). It has been reported that sinonasal intestinal-type adenocarcinoma is closely associated with exposure to hardwood dust, while non-ITAC is considered unrelated to wood dust exposure (Leivo et al. 2021). The average age of onset for non-ITAC is 51 years, and the incidence is slightly lower in males compared to females (1:1.3). The nasal cavity is the most common site of occurrence for non-ITAC, followed by the maxillary sinus and ethmoid sinus (Purgina et al. 2015a). Nasal obstruction, epistaxis, and pain caused by tumor growth and compression of other tissues are common reasons for hospitalization in patients. Based on pathological morphology, non-ITAC can be further divided into low-grade and high-grade non-intestinal-type sinonasal adenocarcinoma. High-grade non-ITAC is rarer, more atypical, and has a poorer prognosis compared to low-grade non-ITAC. Currently, surgery combined with postoperative radiation therapy is considered the standard treatment for non-ITAC (Su et al. 2014). However, high-grade non-ITAC is highly invasive, often presents with atypical clinical symptoms, and is typically diagnosed at an advanced stage. Therefore, adjuvant therapy after surgery and radiation therapy is particularly important. In this article, we report the clinical data of a patient with advanced high-grade non-ITAC who received postoperative radiation therapy followed by immunotherapy and targeted therapy, aiming to provide reference for the treatment of this rare tumor.

## Clinical data

**Fig. 1:**
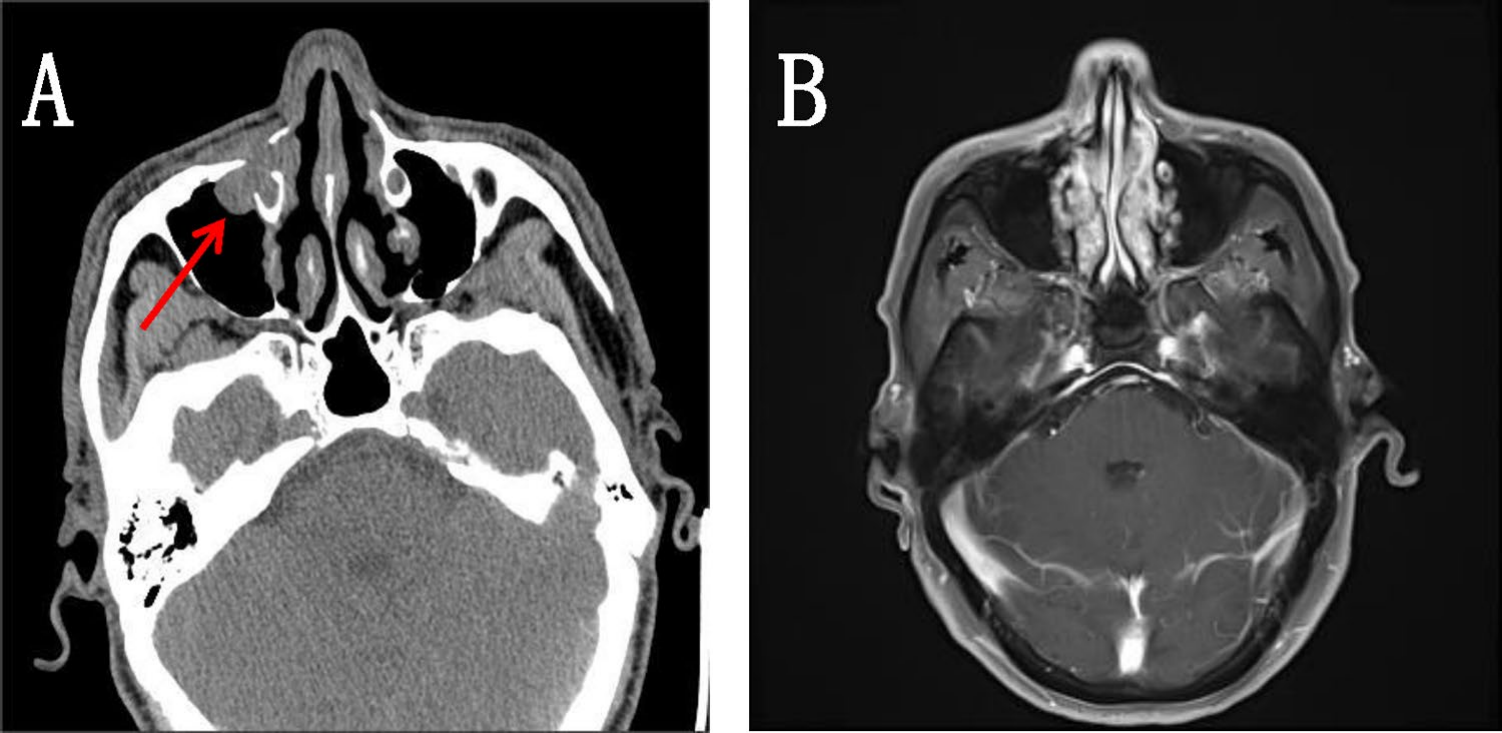
Imaging findings: **A** CT scan of the paranasal sinuses shows a slight soft tissue density shadow in the anterior wall area of the right maxillary sinus with adjacent bone destruction and resorption. The nasal septum is slightly deviated to the right and both nasal turbinates are enlarged. **B** MRI scan of the head shows: 1. Local absence of the anterior wall of the right maxillary sinus, no abnormal enhancement seen after contrast administration

**Fig. 2:**
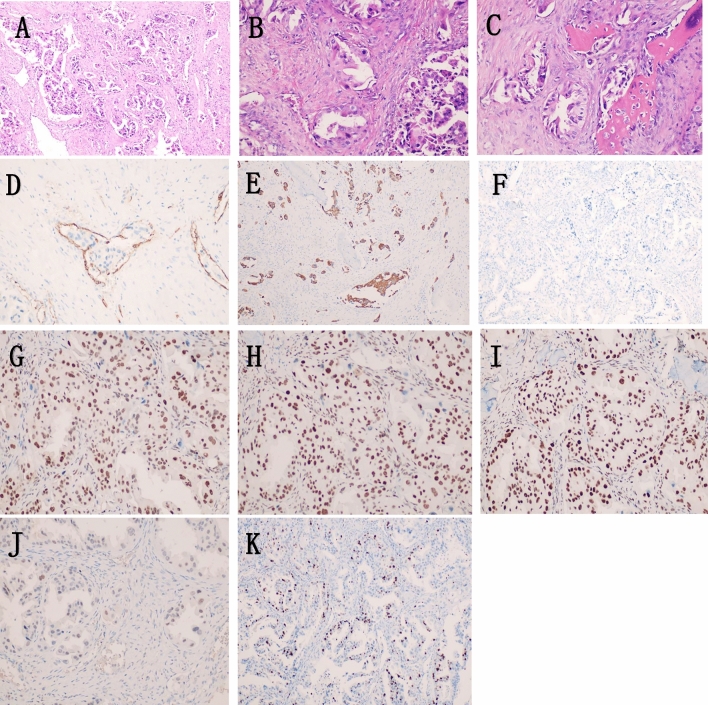
Pathological findings: The tumor demonstrates an infiltrative growth pattern, with tumor cells arranged in glandular and papillary structures, partially organized in small nests. Individual tumor cells can be seen infiltrating the fibrous stroma (**A** HE × 100). The glands are composed of single-layer ciliated columnar cells. The tumor cells are oval or short spindle-shaped, with an increased nuclear-cytoplasmic ratio, eosinophilic cytoplasm, and significant nuclear atypia, manifesting as irregular nuclear shapes, dark staining, vesicular nuclei, prominent nucleoli in some cells, and visible mitotic figures (**B**: HE × 200), no significant necrosis is observed. The tumor stroma exhibits fibrous tissue hyperplasia with a small amount of lymphocytic infiltration, and the tumor is seen invading the surrounding normal bone tissue (**C**: HE × 200). Intravascular cancer emboli are observed, with CD31 staining indicating endothelial cells (**D**: EnVision × 200)。**E**:CK7( +), **F**:CK20(–), **G**:MLH1 ( +)，**H**:MSH2( +)，**I**:MSH6( +), **J**:PMS2( +), **K**:Ki67(+ , 20–30%)

**Fig. 3:**
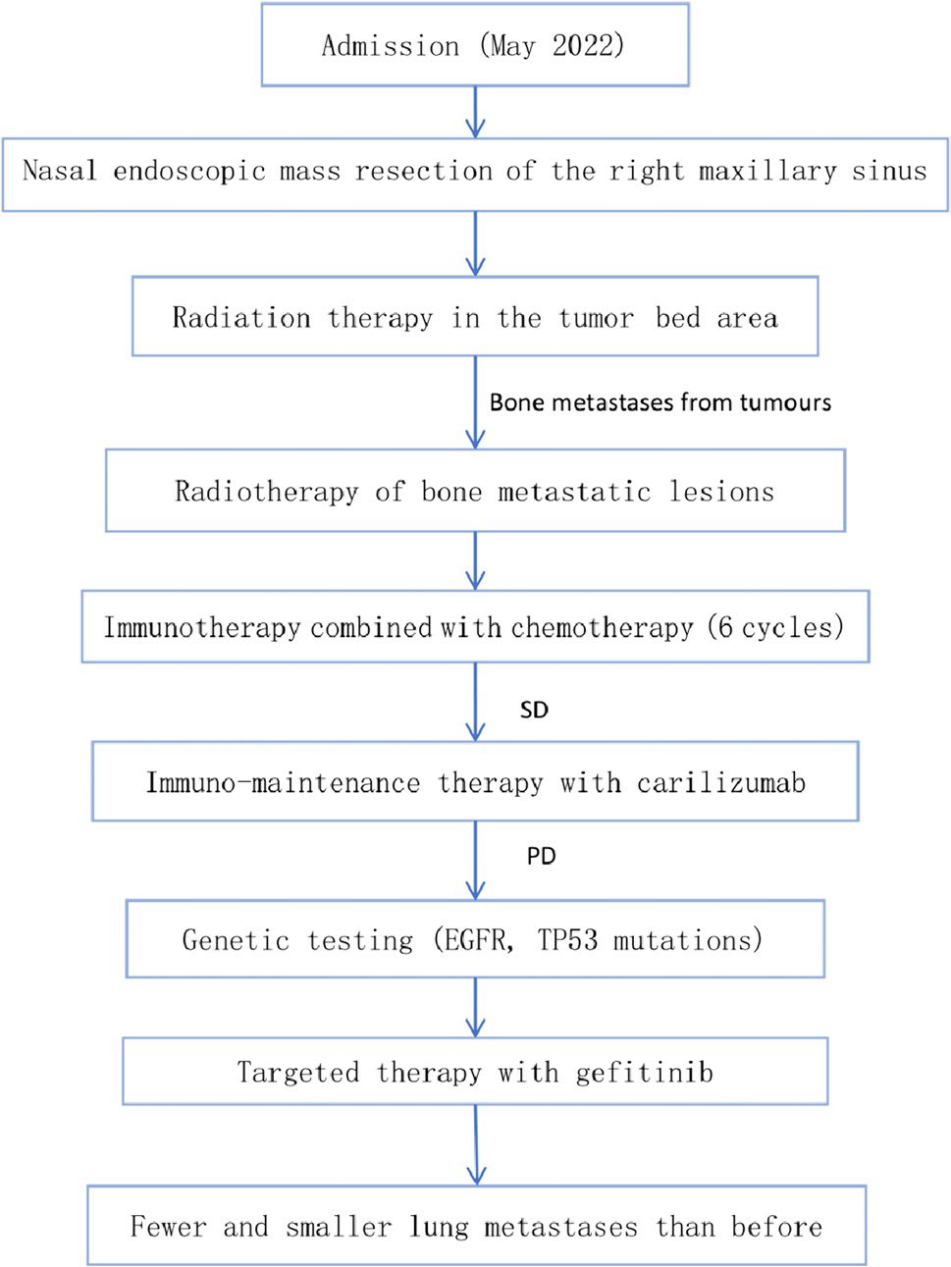
Diagnosis and treatment flowchart

Patient, male, 65 years old, was admitted to our hospital on 5 May 2022 with a chief complaint of “pain in the right maxillary sinus”. Physical examination on admission: slight swelling on the right side of the nasal cheek, no redness or swelling of the skin, palpable subcutaneous hard mass, no mobility, slight tenderness to pressure, chronic nasal congestion on both sides, no evidence of neoplasm or foreign body in the nasal cavity, slightly deviated nasal septum, bilateral hypertrophy of the inferior turbinate. Enhanced computed tomography (CT) scan of the paranasal sinuses showed a mild soft tissue density shadow in the anterior wall of the right maxillary sinus with adjacent bone destruction and resorption, mild deviation of the nasal septum, and bilateral hypertrophy of the nasal turbinate (Fig. 1A). An extended chest CT scan (4 May 2022) showed: 1. Multiple small nodules in both lungs, suggestive of pneumoconiosis or infectious lesions; 2. Enlarged mediastinal lymph nodes and mild bilateral pleural thickening. On 7 May 2022, the patient underwent endoscopic-assisted excision of the mass in the right maxillary sinus. Post-surgical pathology revealed an infiltrative growth pattern of the tumor, with tumor cells arranged in glandular and papillary structures, partially organized in small nests, and individual tumor cell infiltration observed in the fibrous stroma. The glands were composed of single-layer ciliated columnar cells. The tumor cells were oval or short spindle-shaped, with an increased nuclear-cytoplasmic ratio, eosinophilic cytoplasm, and significant nuclear atypia, manifesting as irregular nuclear shapes, dark staining, vesicular nuclei, prominent nucleoli in some cells, and visible mitotic figures. No significant necrosis was observed. Fibrous tissue hyperplasia with a small amount of lymphocytic infiltration was present in the tumor stroma, and the tumor was seen invading the surrounding normal bone tissue. Cancer emboli were detected within the blood vessels. Immunohistochemical staining showed CK7 (+), CK20 ( – ), CDX2 ( – ), SATB2 ( – ), MUC2 ( – ), MLH1 ( +), MSH2 ( +), MSH6 (+), PMS2 (+), Ki67 (+ , 20–30%). Based on the morphological and immunohistochemical findings, the diagnosis of high-grade non-intestinal-type adenocarcinoma was established (Fig. 2). One month after surgery, a follow-up magnetic resonance imaging (MRI) of the head (7 June 2022) showed a local absence of the anterior wall of the right maxillary sinus without abnormal enhancement after contrast administration (Fig. 1B). Based on the auxiliary examinations, radiotherapy was administered to the tumor bed and lymphatic drainage area 1 month after surgery. During the course of radiotherapy (19 July 2022), the patient developed back pain. Subsequent bone scan showed osteolytic bone destruction and abnormal bone metabolism in the right scapula, T8 vertebra and left skeletal bone, suggesting bone metastasis. Palliative radiotherapy was administered to the bone metastatic lesions with a prescription dose of 30 GY/10f. After radiotherapy, the pain improved significantly and six cycles of immunocombination chemotherapy were administered from 2022-10-11 to 2023-02-06, the specific regimen was carilizumab + docetaxel + cisplatin. Stable disease (SD) after five cycles of chemotherapy combined with immunotherapy post assessment, and then continued with carilizumab immuno-maintenance therapy. Review of head and chest CT (2023-05-23): 1. Localized increased bone density in the anterior wall of the right superior collateral sinus. 2. Scattered multinodular nodular shadows in both lungs along the lung texture, considering lung metastases, which were increased and enlarged compared with the previous ones. 3. Bone destruction was seen in the thoracic 8 and 11 vertebrae and accessories, and the splenic bone of the right shoulder, with increased bone density in the left division of the cervical 5 vertebrae, considering metastasis, and the cervical 5 vertebrae was the new lesion. In combination with the adjuvant examination, progressive disease (PD) was considered. Genetic testing showed EGFR and TP53 mutations, and gefitinib targeted therapy was started on 2023-06-01. 2023-08-11 Repeat Head and Chest CT: 1. Localized increased bone density in the anterior wall of the right frontal sinus, unchanged compared to previous. 2. Multiple scattered spots and nodules of varying sizes in both lungs, suggestive of lung metastases. Decreased in number and size compared to previous images. 3. Increased bone density in multiple vertebrae and the right shoulder blade and spleen bone, suggestive of metastases. 2023-11-21 Repeat CT: Multiple small nodular opacities in both lungs, similar to previous findings. The patient is still undergoing oral treatment with gefitinib targeted therapy (Fig. 3).

## Discussion

Primary sinonasal adenocarcinoma is a rare tumor, accounting for less than 1% of all head and neck cancers (Sarradin et al. 2018). In the 5th edition of the World Health Organization (WHO) classification, primary sinonasal adenocarcinoma is divided into two subtypes: intestinal-type sinonasal adenocarcinoma (ITAC) and non-intestinal-type sinonasal adenocarcinoma (non-ITAC) (Thompson et al. 2022). Non-ITAC is an extremely rare adenocarcinoma that lacks both intestinal and salivary features (Purgina et al. 2015a).Non-ITAC can be classified as low-grade or high-grade non-ITAC, the low-grade non-ITAC appears as a well-defined mass with heterogeneous enhancement on contrast-enhanced CT or MRI, High-grade non-ITAC may have more aggressive features such as invasion of adjacent structures, destruction of bony structures and lymphadenopathy. Imaging findings alone may not be sufficient to make a definitive diagnosis and histopathological examination is required. Low-grade non-ITAC has a specific subset of mucinous adenocarcinoma with focal fusion growth or infiltration (El-Naggar et al. 2017). High-grade non-ITAC is even rarer and has rarely been reported in the national and international literature. It is an invasive tumor with low differentiation, marked atypia and greater tissue diversity. Most high-grade non-ITACs present as solid sheet-like structures, sometimes accompanied by scattered glandular or papillary growth, with fewer glandular lumens, which may not be obvious or may appear as small vacuoles (Stelow et al. 2011). Tumor cells are of moderate size with clear or slightly acidophilic cytoplasm. They show high pleomorphism, increased mitotic activity and may show signs of necrosis. They infiltrate the surrounding soft and bone tissues and often involve blood vessels and nerves (Agaimy et al. 2021). High-grade non-ITAC may have many overlapping features with other malignancies in this region. Therefore, pathologists should strive to exclude other tumours before making this diagnosis. In addition, due to the morphological heterogeneity of the lesion, it is important to consider the possibility of metastatic malignancy (Stelow et al. 2011 Jul).Low-grade non-ITAC often stains positive for S100 protein, SOX10, and DOG1, whereas high-grade non-ITAC may show immunohistochemical changes. High-grade non-ITAC may also express focal neuroendocrine markers (Purgina et al. 2015b). Recently, SATB2 has been identified as a potential diagnostic biomarker that is highly specific for differentiating ITAC from non-ITAC (Skalova et al. 2018). There are few reports on genetic analysis of nasal non-ITAC. It has been reported in the literature that some high-grade non-ITAC may be associated with an ETV6 gene rearrangement, usually an ETV6-NTRK3 gene fusion (Klubíčková et al. 2023 Aug).

The main clinical manifestations of non-ITAC are nasal congestion and epistaxis, and pain caused by tumor growth compressing other tissues. Median survival is 5.5 months (range: 2 months to 5 years), with higher-grade tumours having a worse prognosis than lower-grade tumours (Franchi et al. 2005). High-grade tumours mainly affect the nasal cavity and maxillary sinuses, while low-grade tumours mainly affect the nasal cavity and ethmoid sinuses (Perez-Ordonez 2009; Bracigliano et al. 2021). Due to the rarity of these tumours, their histological diversity and their proximity to important structures such as the orbit, skull base and brain, diagnosis and treatment of these tumours are challenging. Surgery is the first-line treatment for sinonasal adenocarcinoma of the nasal cavity and paranasal sinuses (Meccariello et al. 2016; Nicolai et al. 2011). Research has reported that bilateral ethmoidectomy can minimise the occurrence of secondary tumours, as tumor nests in healthy mucosa away from the tumor have been demonstrated in these regions (Bussi et al. 2002). In recent years, the range of endoscopic procedures has expanded with the development of endoscopic techniques. However, these procedures have mainly focused on smaller and lower stage tumours (Kassam et al. 2005; Gerven et al. 2011). Currently, the gold standard treatment for primary sinonasal adenocarcinoma is surgical removal of the tumor with negative margins followed by radiotherapy, which is suitable for most patients (Poorten and Jorissen 2020). Antognoni P reported a study of 30 cases of intestinal-type sinonasal adenocarcinoma treated with endoscopic resection followed by adjuvant radiotherapy. The retrospective analysis suggested that endoscopic surgery combined with postoperative radiotherapy may be considered a safe, minimally invasive, and highly effective option for the treatment of specific ITAC of the paranasal sinuses (Antognoni et al. 2015). Tachino H reported the first case of successful cure of locally advanced low-grade non-intestinal-type sinonasal adenocarcinoma in a patient treated with concurrent chemoradiotherapy, achieving complete pathological and clinical remission. This study suggested that the regimen of super-selective intra-arterial injection of cisplatin (CDDP) combined with conventional fractionated radiotherapy followed by salvage surgery may be beneficial for the treatment of sinonasal adenocarcinoma (Tachino et al. 2020). Bossi compared the prognosis of two groups of patients: one group received postoperative radiotherapy (group A) and the other group received platinum-based induction chemotherapy followed by standard treatment (group B). The study found that the 5-year overall survival (OS) rate was 42% in group A and 70% in group B (P = 0.041), and the 5-year disease-free survival (DFS) rate was 40% in group A and 66% in group B (P = 0.009). These results suggest that preoperative induction chemotherapy may be beneficial for the prognosis of ITAC (Bossi et al. 2013).

The standard of care for advanced non-ITAC sinus cancer remains radical surgery and adjuvant radiotherapy using a multimodality approach. Advances in imaging, surgical and endoscopic techniques, radiotherapy techniques (intensity modulated radiotherapy, volumetric modulated arc therapy, and heavy particle beam radiotherapy) and strategies involving neoadjuvant chemotherapy have shown promising results in improving prognosis(Leivo et al. 2021). However, the prognosis for advanced non-ITAC sinus cancer remains poor. To assess the prognosis of sinonasal adenocarcinoma, Veuger J conducted a systematic review analysing 21 studies investigating the impact of biomarkers on the prognosis of sinonasal non-ITAC. The study found that expression of the mucin antigen sialosyl-Tn, C-erbB-2 tumor protein, TIMP3, TP53, vascular endothelial growth factor, ANXA2, MUC1 and histological subtypes of mucinous tissue had a significant negative impact on survival. A comprehensive understanding of the biomarkers associated with ITAC/non-ITAC prognosis may provide therapeutic targets to improve treatment strategies (Veuger et al. 2023).

Immunotherapy, which harnesses the immune system to recognise and eliminate cancer, has become one of the mainstays of cancer treatment. In the past decade, immunotherapy has significantly improved survival in many cancer patients (Dagher et al. 2023). In a study of 30 cases of high-grade sinonasal cancer, including 2 cases of non-ITAC, they examined the expression of major histocompatibility complex molecules, leukocyte infiltration and chemokine expression. They found that the chemokines CXCL8 and CXCL5 were up-regulated in high-grade sinonasal cancer, affecting leukocyte activation and trafficking, angiogenesis, metastasis and cancer cell proliferation. On the other hand, some chemokines such as CCL28 and CCL14 were downregulated in high-grade neuroendocrine carcinoma compared to normal tissue. Targeting migration-associated chemokines and their receptors in sinonasal tumours may be beneficial for immunotherapy (Bell et al. 2020). Currently, there are no guidelines recommending immunotherapy for high-grade non-ITAC. A large phase II trial of nivolumab and ipilimumab in patients with rare tumours, including squamous cell carcinoma and adenocarcinoma of the sinonasal region (NCT02834013), is underway and is expected to provide more detailed and accurate descriptions of treatment outcomes.

We reported for the first time a case of high-grade non-ITAC of maxillary sinus origin treated with postoperative radiotherapy followed by immunocombination chemotherapy and then targeted therapy. The patient was clinically diagnosed as high-grade non-ITAC of the maxillary sinus with cervical lymph node and bone metastasis, and the patient was treated with local radiotherapy and then with 6 cycles of “immunocombination chemotherapy” protocol after endoscopic resection of the tumor. The specific regimen of which was carilizumab + docetaxel + cisplatin. The immuno-maintenance therapy of carilizumab was continued until the tumor progressed one year after the operation (new bone metastatic foci and suspected lung nodal foci increased in size, and tumor metastases were taken into consideration), and the genetic test showed mutations of EGFR and TP53, and the targeted therapy of gefitinib was given. After treatment, the metastatic foci in the lungs were smaller than before. Currently, the disease is in a stable state.

## Conclusion

Non-intestinal-type sinonasal adenocarcinoma is a rare malignancy of the nasal cavity and paranasal sinuses. It is distinct from the more common intestinal-type sinonasal adenocarcinoma and lacks both intestinal and salivary features. High-grade non-ITAC is particularly rare and has aggressive features on histopathology and imaging. Treatment typically involves surgical resection, with adjuvant therapy considered in certain cases. Prognosis can vary depending on several factors and long-term outcomes are not well established. Immunotherapy and targeted therapy may offer potential treatment options for high-grade non-intestinal adenoid cystic carcinoma of the maxillary sinus.

## Data Availability

The original contributions presented in the study are included in the article. Further inquiries can be directed to the corresponding author.

## References

[CR1] Agaimy A, Mueller SK, Bishop JA, Chiosea SI (2021) Primary and secondary/ metastatic salivary duct carcinoma presenting within the sinonasal tract. Head Neck Pathol 15:769–779. 10.1007/s12105-020-01271-8 33428064 10.1007/s12105-020-01271-8 PMC8384981

[CR2] Antognoni P, Turri-Zanoni M, Gottardo S, Molteni M, Volpi L, Facco C, Freguia S, Mordacchini C, AlQahtani A, Bignami M, Capella C, Castelnuovo P (2015) Endoscopic resection followed by adjuvant radiotherapy for sinonasal intestinal-type adenocarcinoma: retrospective analysis of 30 consecutive patients. Head Neck 37(5):677–684. 10.1002/hed.23660 24596075 10.1002/hed.23660

[CR3] Bell D, Bell A, Ferrarotto R, Glisson B, Takahashi Y, Fuller G, Weber R, Hanna E (2020) High-grade sinonasal carcinomas and sur-veillance of differential expression in immune related transcriptome. Ann Diagn Pathol 49:151622. 10.1016/j.anndiagpath.2020.151622 32927372 10.1016/j.anndiagpath.2020.151622

[CR4] Bossi P, Perrone F, Micel R, Cantu G, Mariani L, Orlandi E et al (2013) Tp53 status as guide for the management of ethmoid sinus intestinal-type adenocarcinoma. Oral Oncol 49:413–419. 10.1016/j.oraloncology.2012.12.011 23369851 10.1016/j.oraloncology.2012.12.011

[CR5] Bracigliano A, Tatangelo F, Perri F, Di Lorenzo G, Tafuto R, Ottaiano A, Clemente O, Barretta ML, Losito NS, Santorsola M, Tafuto S (2021) Malignant sinonasal tumors: update on histological and clinical management. Curr Oncol 28(4):2420–2438. 10.3390/curroncol28040222 34287240 10.3390/curroncol28040222 PMC8293118

[CR6] Bussi M, Gervasio CF, Riontino E (2002) Study of ethmoidal mucosa in a population at occupational high risk of sinonasal adenocarcinoma. Acta Otolaryngol 122(02):197–201 11936913 10.1080/00016480252814225

[CR7] Dagher OK, Schwab RD, Brookens SK, Posey AD Jr (2023) Advances in cancer immunotherapies. Cell 186(8):1814-1814.e1. 10.1016/j.cell.2023.02.039 37059073 10.1016/j.cell.2023.02.039

[CR8] El-Naggar AK, Chan JKC, Grandis JR, Takata T, Slootweg PJ (2017) WHO Classification of Head and Neck Tumours. IARC Press, Lyon

[CR9] Franchi A, Santucci M, Wenig BM (2005) Adenocarcinoma. WHO histological classification of tumors of the nasal cavity and paranasal sinuses. In: Barnes L, Eveson JW, Reichardt P, Sidransky D, editors. Pathology and genetics of head and neck tumors. Lyon: IARC Press; p. 20–23

[CR10] Kassam A, Snyderman CH, Mintz A et al (2005) Expanded endonasal approach: the rostrocaudal axis. Part I. Crista galli to the sella turcica. Neurosurg Focus. 19(1):3 16078817

[CR11] Klubíčková N, Mosaieby E, Ptáková N, Trinquet A, Laé M, Costes-Martineau V, Skálová A (2023) High-grade non-intestinal type sinonasal adenocarcinoma with ETV6::NTRK3 fusion, distinct from secretory carcinoma by immunoprofile and morphology. Virchows Arch 483(2):187–195. 10.1007/s00428-023-03587-6 37415052 10.1007/s00428-023-03587-6 PMC10412680

[CR12] Leivo I, Holmila R, Luce D, Steiniche T, Dictor M, Heikkilä P, Husgafvel-Pursiainen K, Wolff H (2021) Occurrence of sinonasal intestinal-type adenocarcinoma and non-intestinal-type adenocarcinoma in two countries with different patterns of wood dust exposure. Cancers (Basel) 13(20):5245. 10.3390/cancers13205245 34680393 10.3390/cancers13205245 PMC8533857

[CR13] Meccariello G, Deganello A, Choussy O et al (2016) Endoscopic nasal versus open approach for the management of sinonasal adenocarcinoma: a pooled-analysis of 1826 patients. Head Neck 38(S1):E2267–E2274 26335008 10.1002/hed.24182

[CR14] Nicolai P, Villaret AB, Bottazzoli M et al (2011) Ethmoid adenocar- cinoma–from craniofacial to endoscopic resections: a single- institution experience over 25 years. Otolaryngol Head Neck Surg 145(2):330–337 21515803 10.1177/0194599811403873

[CR15] Perez-Ordonez B (2009) Hamartomas, papillomas and adenocarcinomas of the sinonasal tract and nasopharynx. J Clin Pathol 62(12):1085–1095 19946095 10.1136/jcp.2007.053702

[CR16] Poorten VV, Jorissen M (2020) A comprehensive update on intestinal- and non-intestinal-type adenocarcinomas. Adv Otorhinolaryngol 84:137–153. 10.1159/000457934 32731230 10.1159/000457934

[CR17] Purgina B, Bastaki JM, Duvvuri U, Seethala RR (2015a) A subset of sinonasal non-intestinal type adenocarcinomas are truly sero-mucinous adenocarcinomas: a morphologic and immunophenotypic assessment and description of a novel pitfall. Head Neck Pathol 9:436–446. 10.1007/s12105-015-0615-3 25690258 10.1007/s12105-015-0615-3 PMC4651926

[CR18] Purgina B, Bastaki JM, Duvvuri U, Seethala RR (2015b) A subset of sinonasal non-intestinal type adenocarcinomas are truly seromucinous adenocarcinomas: a morphologic and immunophenotypic assessment and description of a novel pitfall. Head Neck Pathol 9:436–446 25690258 10.1007/s12105-015-0615-3 PMC4651926

[CR19] Sarradin V, Siegfried A, Uro-Coste E et al (2018) Classification de l‘OMS 2017 des tumeurs de la Tete et du comericasles nou- veautes et mise a jour des methodes diagnostiques [WHO classification of head and neck tumours 2017: main novelties and update of diagnostic methods]. Bull Cancer. 105(6):596–602 29759330 10.1016/j.bulcan.2018.04.004

[CR20] Skalova A, Sar A, Laco J et al (2018) The role of SATB2 as a diagnostic marker of sinonasal intestinal-type adenocarcinoma. Appl Immunohistochem Mol Morphol 26(2):140–146. 10.1097/PAI.0000000000000388 27258560 10.1097/PAI.0000000000000388

[CR22] Stelow EB, Jo VY, Mills SE, Carlson DL (2011) A histologic and immunohistochemical study describing the diversity of tumors classified as sinonasal high-grade nonintestinal adenocarcinomas. Am J Surg Pathol 35:971–980. 10.1097/PAS.0b013e31821cbd72 21677536 10.1097/PAS.0b013e31821cbd72

[CR23] Su SY, Kupferman ME, Demonte F, Levine NB, Raza SM, Hanna E (2014) Endoscopic resection of sinonasal cancers. Curr Oncol Rep 16:1–8. 10.1007/s11912-013-0369-6 10.1007/s11912-013-0369-6 24445501

[CR24] Tachino H, Takakura H, Shojaku H, Fujisaka M, Akaogi K, Kawabe H, Naruto N, Shojaku H, Noguchi K, Miwa S, Imura J, Maeda Y (2020) Case report: response to intra-arterial cisplatin and concurrent radiotherapy followed by salvage surgery in a patient with advanced primary sinonasal low-grade non-intestinal adenocarcinoma. Front Surg 10(7):599392. 10.3389/fsurg.2020.599392. 10.3389/fsurg.2020.599392 PMC7758204 33363200

[CR25] Thompson LDR, Loney EL, Bishop JA, et al (2022) WHO Classification of Tumours Editorial Board. Head and neck tumours. [Internet; beta version ahead of print] Lyon (France): International Agency for Research on Cancer; Chapter 2: Nasal, paranasal, and skull base tumours

[CR26] Van Gerven L, Jorissen M, Nuyts S, Hermans R, Vander PV (2011) Long-term follow-up of 44 patients with adenocarcinoma of the nasal cavity and sinuses primarily treated with endoscopic resection followed by radiotherapy. Head Neck 33(06):898–904 20967864 10.1002/hed.21556

[CR27] Veuger J, Kuipers NC, Willems SM, Halmos GB (2023) Tumor markers and their prognostic value in sinonasal ITAC/Non-ITAC. Cancers (Basel) 15(12):3201. 10.3390/cancers15123201.PMID:37370810;PMCID:PMC10296805 37370810 10.3390/cancers15123201 PMC10296805

